# Human oncoprotein Musashi-2 N-terminal RNA recognition motif backbone assignment and identification of RNA-binding pocket

**DOI:** 10.18632/oncotarget.22540

**Published:** 2017-11-20

**Authors:** Lan Lan, Minli Xing, Justin T. Douglas, Philip Gao, Robert P. Hanzlik, Liang Xu

**Affiliations:** ^1^ Departments of Molecular Biosciences, The University of Kansas, Lawrence, KS, USA; ^2^ Bio-NMR Core Facility, The University of Kansas, Lawrence, KS, USA; ^3^ Protein Production Group, NIH COBRE in Protein Structure and Function, The University of Kansas, Lawrence, KS, USA; ^4^ Department of Medicinal Chemistry, The University of Kansas, Lawrence, KS, USA; ^5^ Department of Radiation Oncology, The University of Kansas Cancer Center, Kansas City, KS, USA

**Keywords:** RNA-binding protein, RNA-binding pocket, nuclear magnetic resonance, backbone assignment, Musashi

## Abstract

RNA-binding protein Musashi-2 (MSI2) is a key regulator in stem cells, it is over-expressed in a variety of cancers and its higher expression is associated with poor prognosis. Like Musashi-1, it contains two N-terminal RRMs (RNA-recognition Motifs, also called RBDs (RNA-binding Domains)), RRM1 and RRM2, which mediate the binding to their target mRNAs. Previous studies have obtained the three-dimensional structures of the RBDs of Musashi-1 and the RBD1:RNA complex. Here we show the binding of MSI2-RRM1 to a 15nt *Numb* RNA in Fluorescence Polarization assay and time resolved Fluorescence Resonance Energy Transfer assay. Using nuclear magnetic resonance (NMR) spectroscopy we assigned the backbone resonances of MSI2-RRM1, and characterized the direct interaction of RRM1 to *Numb* RNA r(GUAGU). Our NMR titration and structure modeling studies showed that MSI2-RRM1 and MSI1-RBD1 have similar RNA binding events and binding pockets. This work adds significant information to MSI2-RRM1 structure and RNA binding pocket, and contributes to the development of MSI2 specific and MSI1/MSI2 dual inhibitors.

## INTRODUCTION

RNA-binding proteins (RBPs) are important regulators of mRNA stability and translation, and dysregulation of RBPs is implicated in many disease conditions including cancer. The RNA-binding protein Musashi-2 (MSI2) is one of the Musashi family of RNA-binding proteins that plays redundant as well as independent roles as Musashi-1 (MSI1) in stem cells [1–4]. MSI2 has recently been found to be over-expressed in many cancers, including hematologic malignancies [5–11], colorectal adenocarcinomas [12–14], lung [15], pancreatic cancers [16–18], and glioblastoma [19]. It maintains cancer stem cell populations and regulates cancer invasion, metastasis and development of more aggressive cancer phenotypes, including drug resistance, by mediating mRNA stability and translation of proteins that involve in oncogenic pathways (summarized in [20–22]). Overexpression of MSI2 has led to drug resistance in ovarian cancer cells [23], promotes invasion and metastasis in colorectal cancer, non-small cell lung cancer and pancreatic cancer [14, 15, 17]. Knocking down of MSI2 sensitizes cancer cells to treatment in ovarian cancer cells and in acute myeloid leukemia [23, 24], MSI2 depletion stimulates an epithelial to mesenchymal phenotype [15]. These findings suggest that MSI2 is a promising therapeutic target for cancer.

Both Musashi proteins belong to the class A/B heterogeneous nuclear ribonucleoproteins (hnRNPs). They each have two N-terminal RRMs (RNA-recognition Motifs, also called RBDs (RNA-binding Domains)), RRM1 and RRM2, which mediate the binding to their target mRNAs [3]. Like MSI1, MSI2 post-transcriptionally regulates mRNAs by binding to the recognition motifs located at 3′-UTR of target mRNAs. One of the motifs, r(UAG), was shared between MSI1 and MSI2 [25, 26]. The residues that recognize r(UAG) in MSI1 are highly conserved between MSI1 and MSI2 [26].

MSI1 and MSI2 share several overlapping targets that are involved in oncogenic pathways (summarized in [20, 22]). One of the targets is *Numb* [6, 7, 27, 28], a negative regulator of the Notch signaling. MSI proteins bind to *Numb* mRNA and inhibits its translation, leading to elevated Notch signaling, increased proliferation and survival, and decreased apoptosis of cancer cells. Specifically, Ito *et al.* reported in Nature that the Musashi-*Numb* pathway can control the differentiation of Chronic myelogenous leukemia (CML); expression of *Numb* as a result of MSI2 loss impairs the development and propagation of blast crisis CML *in vitro* and *in vivo* [6].

The solution structures of the two N-terminal RBDs of mouse Msi1 and their interactions with RNA have been studied extensively [25, 26, 29, 30]. The two RBDs of mouse Msi1 share the same ribonucleoprotien (RNP)-type fold, and MSI1-RBD1 can specifically bind to RNA by stacking interactions between aromatic residues and RNA bases. However, no study to date has examined Msi2/MSI2 residues that directly interact with RNA, and there are no high-resolution Msi2/MSI2 RRMs structures available. Thus, investigation of how RRMs of MSI2 interact with RNA can contribute to identifying novel compounds that can disrupt these unique MSI2-RNA interactions. Currently, there are three studies including ours in developing small-molecule inhibitors of MSI. The inhibitors identified from these studies have Ki values ranging from ∼0.5 µM to 5 µM [31–33], yet the most potent one (–)-gossypol (Ki∼0.5 µM) from our study is not MSI specific [33]. With the help of structure-based rational design based on NMR structure, we will develop more potent and specific MSI inhibitors.

Here we characterize MSI2-RRM1 and RNA interactions by FP (florescence polarization), TR-FRET (time resolved Fluorescence Resonance Energy Transfer). We also describe an NMR (Nuclear Magnetic Resonance Spectroscopy) investigation of backbone assignment of MSI2-RRM1 and its intermolecular interactions with RNA. Based on these studies, we identified the RNA-binding pocket of MSI2-RRM1, and revealed the similarities between the binding pockets of MSI2-RRM1 and MSI1-RBD1.

## RESULTS

### Musashi-2-Numb RNA binding

**Figure 1:**
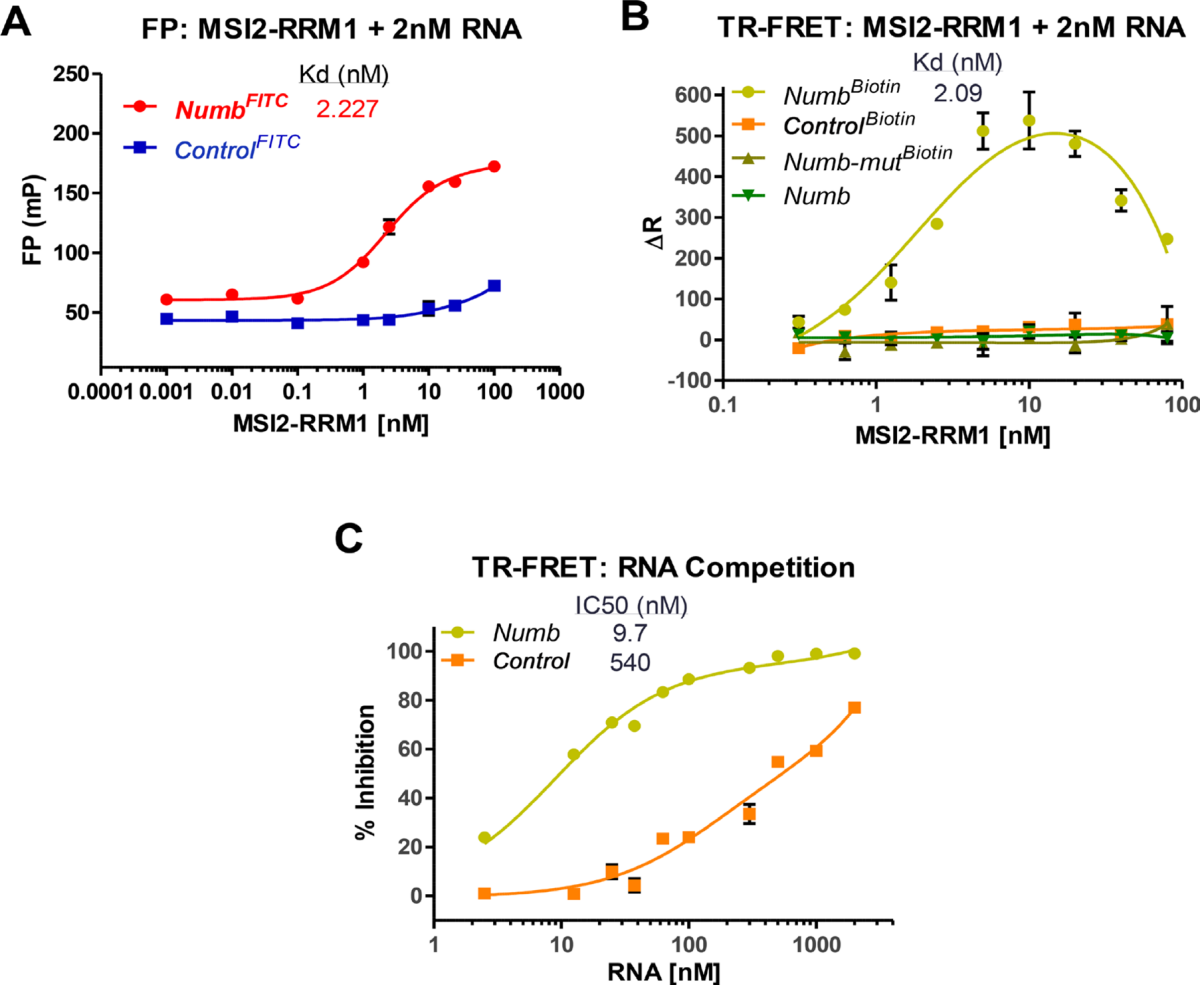
MSI2-RRM1 binds to *Numb* RNA (**A**) Binding between RNA Recognition Motif 1 (aa 21-111) of MSI2 (MSI2-RRM1) to *Numb* RNA (*Numb*^*FITC*^: 5′-UAGGUAGUAGUUUUA-FITC-3′), but not to *Control*^*FITC*^ in FP assay. The concentration of FITC tagged-RNA used in the assay is 2 nM. (*n* > 3) (**B**) Binding between MSI2-RRM1 to *Numb* RNA (*Numb*^*Biotin*^), but not to *Numb-mut*^*Biotin*^ *, Control*^*Biotin*^, or non-labelled *Numb* in TR-FRET assay. The concentration of RNA used in the assay is 2 nM. (*n* > 3) (**C**) Increasing concentrations of unlabeled *Numb* or *Control* RNAs were added to the preformed *Numb*^*Biotin*^-MSI2-RRM1 complexes in TR-FRET. *Numb* can displace *Numb*^*Biotin*^. Displacement of *Numb*^*Biotin*^ at high concentrations of *Control* RNA are due to non-specific binding. (*n* > 3).

Here we show that the *Numb*^*FITC*^ but not a *Control* RNA (*Control*^*FITC*^) with a scrambled sequence binds to MSI2-RRM1, as indicated by the increase FP value (Figure 1A). Such binding is also evident in TR-FRET assay (Figure 1B). Using biotinylated *Numb* RNA (*Numb*^*Biotin*^: 5′UAGGUAGUAGUUUUA-Biotin), the TR-FRET assay detects a Kd of 2.07 nM, whereas a *Control* RNA (*Control*^*Biotin*^), a *Numb* mutant (*Numb-mut*^*Biotin*^: 5′UAGCAUCAUCAUUUA-Biotin) or a non-labelled *Numb* (Figure 1B) show no detectable binding in the nM range. A competition assay on preformed MSI2-RRM1-*Numb*^*Biotin*^ complex indicates that only *Numb* but not *Control* RNA can displace *Numb*^*Biotin*^ (Figure 1C).

### Backbone assignment

To keep the amino acid numbers of recombinant MSI2-RRM1 consistent with those of MSI2, residues of the recombinant MSI2-RRM1 are numbered starting from negative 3. In other words, the first residue of the N-terminal hexahistidine tag is M-3, and K111 is the C-terminal end. Residues M-3 to A20 comprise the N-terminal hexahistidine tag and TEV protease recognition site. G21 is the first residue found in MSI2-RRM1, and it corresponds to G21 in MSI2 sequence (Figure 5A).

**Figure 2:**
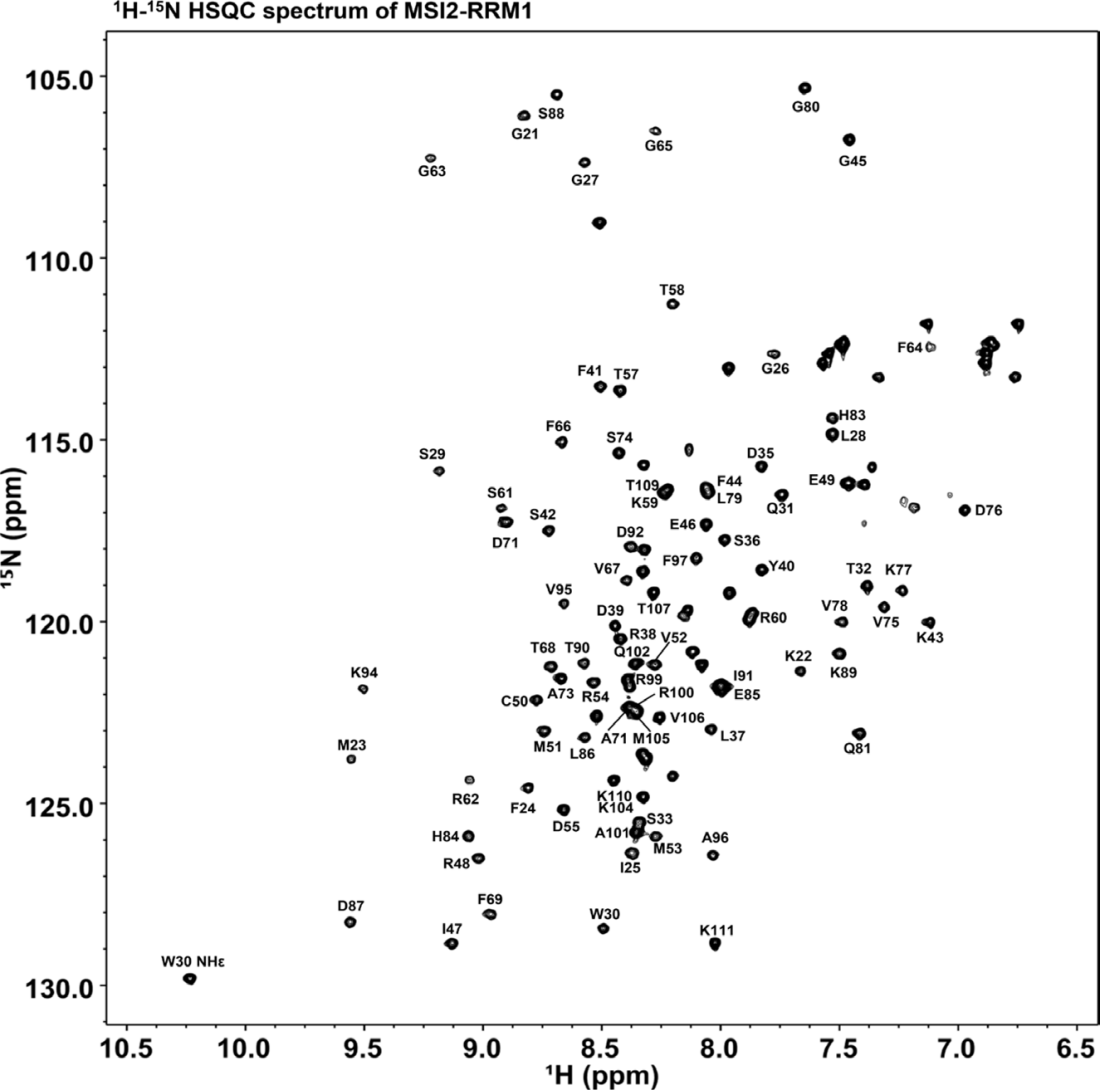
^1^H-^15^N HSQC spectrum of ^15^N labeled MSI2-RRM1 The assignments of G21-K111 backbone (^1^H, ^15^NH) and W30 side chain (^1^Hε, ^15^Nε) are annotated.

**Figure 3:**
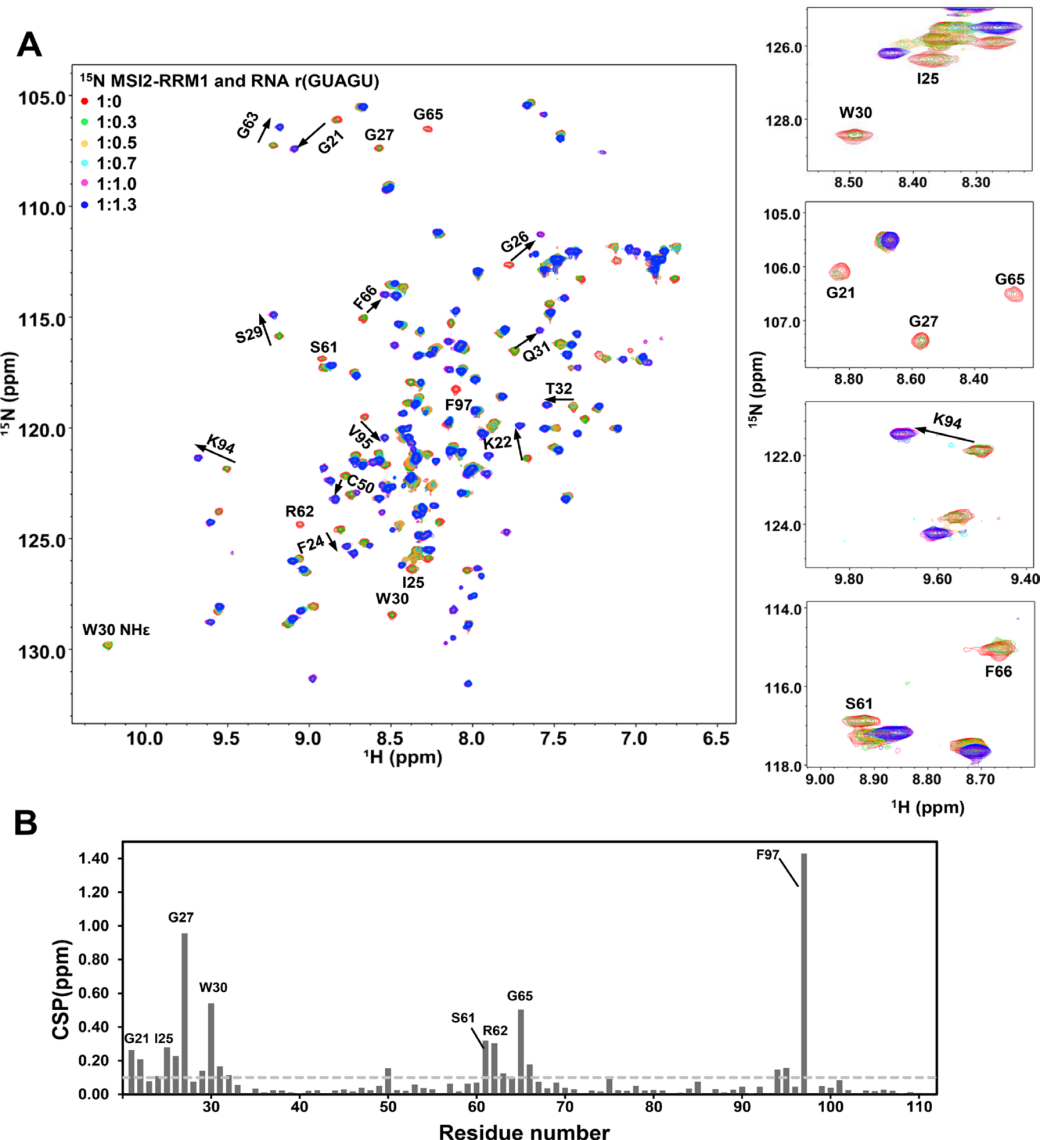
NMR titration of ^15^N labeled MSI2-RRM1 with *Numb5* (**A**) Overlay of ^1^H-^15^N HSQC spectra of MSI2-RRM1 (80 µM) in the presence of 0 (red), 0.3(green), 0.5 (orange), 0.7 (cyan), 1.0 (magenta), and 1.3 (blue) molar equivalent of *Numb5*. Representative peaks showing large CSPs are annotated. (**B**) Weighted CSPs for backbone ^1^H and ^15^NH resonances of MSI2-RRM1 when titrated with 1:1 molar equivalent of *Numb5*. Weighted CSPs were calculated by the equation: Δδ = [((ΔδH)^2^ + (ΔδN/5)^2^)/2]^1/2^. The value of average Δδ plus one standard deviation is 0.1 (indicated by the horizontal dashed line). Δδ values above the horizontal dashed line are significant.

**Figure 4:**
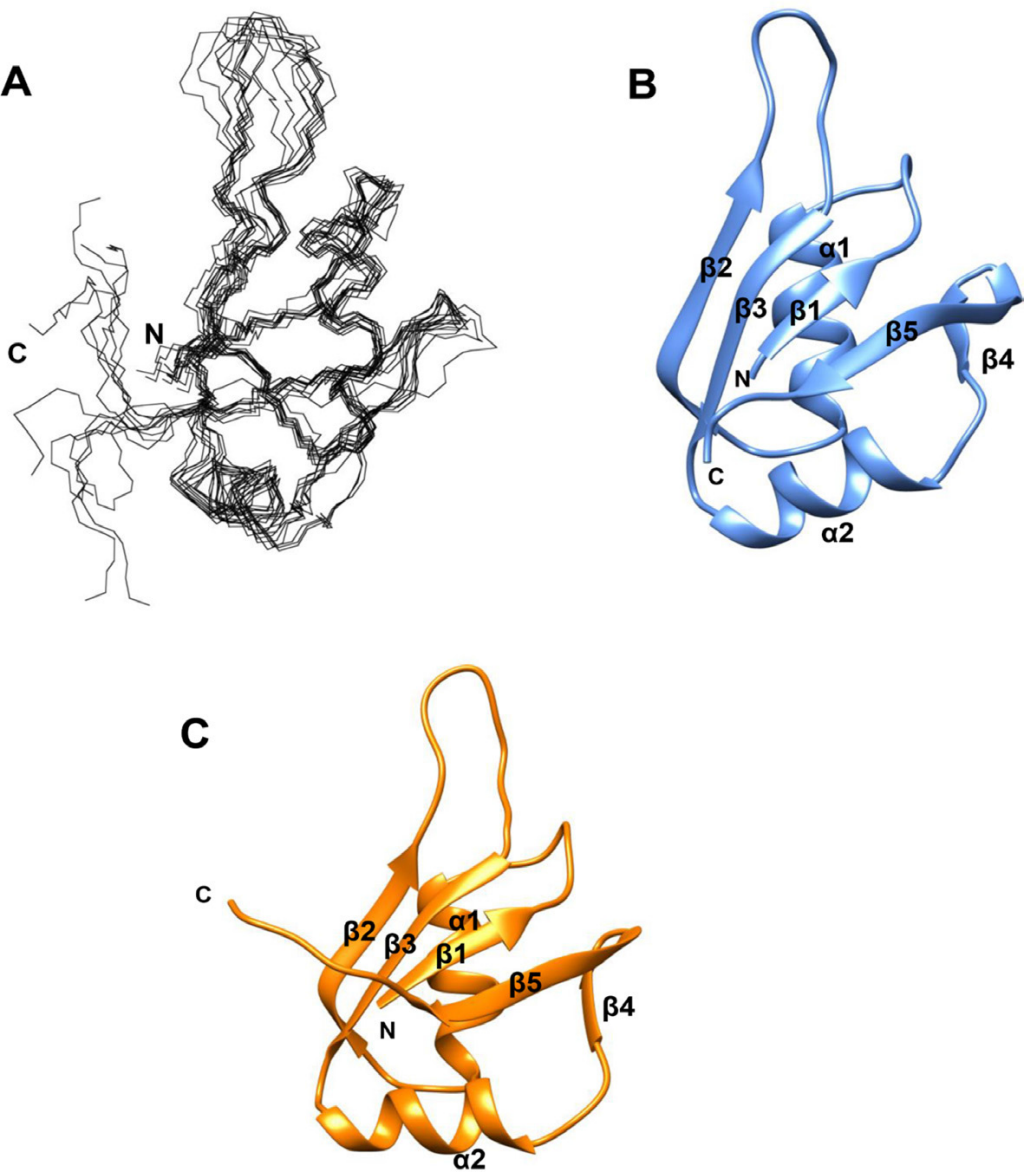
MSI2-RRM1 structure prediction (**A**) Backbone atoms superposition of residues 23–100 for the 10 lowest energy CS-ROSETTA structures of MSI2-RRM1. (**B**) Ribbon diagram representation of the lowest energy CS-ROSETTA structure of MSI2-RRM1. Unstructured regions including residues –3–22 and 101–111 are not shown. The five β-sheets are labeled β1–β5 and the two ɑ-helices are labeled ɑ1 and ɑ2. (**C**) Ribbon diagram representation of the homology model of MSI2-RRM1 (residues 23–100) generated by SWISS-MODEL.

**Figure 5:**
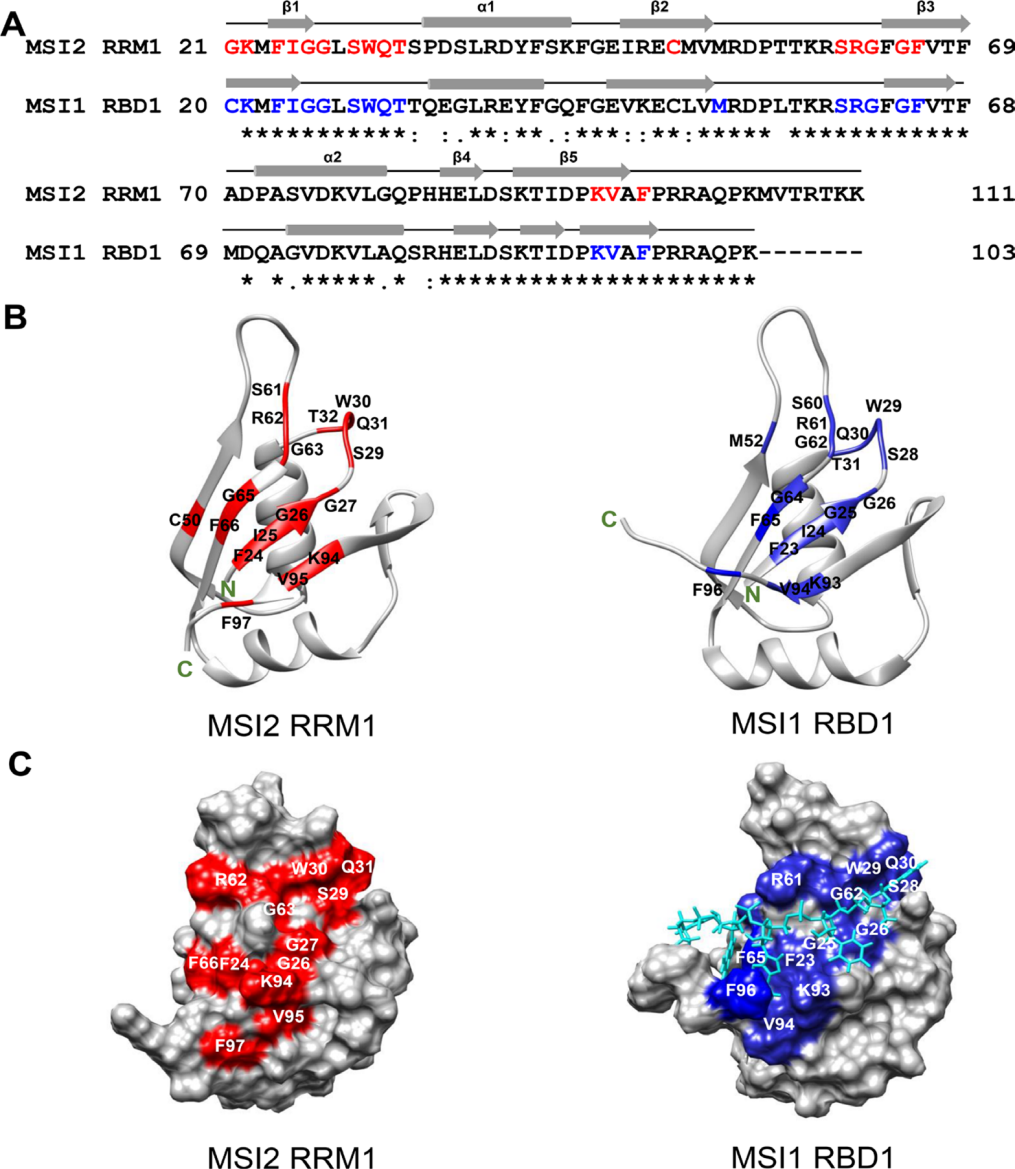
Sequence alignment and CSPs mapping of MSI2-RRM1 and MSI1-RBD1 (**A**) Sequence alignment of MSI2-RRM1 (residues 21–111) and MSI1-RBD1 (residues 20–103). Identical residues are indicated by asterisks, conversed residues by colons and semi-conserved residues by periods. The secondary structure elements in MSI2-RRM1 and MSI1-RBD1 are shown as arrows (β-sheets) and cylinders (α-helices). In MSI2-RRM1, residues with CSPs higher than 0.1 ppm are colored red. In MSI1-RBD1, residues that show pronounced CSPs are colored blue. (**B**) Mapping of CSPs data on to the ribbon diagram representations of the CS-ROSETTA model of MSI2-RRM1 and the NMR structural model of MSI1-RBD1 (PDB 2RS2). (**C**) Mapping of CSPs data on to the surface representations of the CS-ROSETTA model of MSI2-RRM1 and the NMR structural model of MSI1-RBD1 (PDB 2RS2). *Numb5* is colored cyan.

Figure 2 shows the 2D ^1^H-^15^N HSQC spectrum of MSI2-RRM1 annotated with the ^1^H, ^15^NH assignment of residues G21-K111 and W30 side chain NHε. Overall, 92.6% of the non-proline backbone resonances were assigned. Residues not assigned at this time are M-3 through S4 in the hexahistidine tag, and S18 in the TEV protease recognition site. Backbone ^1^H, ^15^N, and ^13^C assignments have been deposited in the BMRB data bank under accession number 27111.

### RNA titration

To map the RNA-binding interface of MSI2-RRM1, we titrated 5 nt *Numb* (*Numb5*: GUAGU) stepwise into ^15^N labeled protein, and recorded a series of 2D ^1^H-^15^N HSQC spectra. Significant perturbations of the chemical shifts of many backbone resonances of MSI2-RRM1 are evident upon binding (Figure 3A). The chemical shift changes reached a plateau at 1:1 molar ratio of MSI2-RRM1:*Numb5*, indicating a 1:1 stoichiometry of MSI2-RRM1 and *Numb5* complex. The peaks of many residues broadened or disappeared at substoichiometirc ratios of MSI2-RRM1:*Numb5*, and reappeared at new positions at 1:1 stoichiometric ratio of MSI2-RRM1:*Numb5.* For some residues, their resonances shifted to nearby positions, while for others, the chemical shifts were perturbed so significantly that the new locations cannot be determined. This behavior may result from direct binding of ligand to protein in which the free and bound states undergo slow exchange in the NMR chemical shift time scale, or ligand induced conformational changes. Chemical shift perturbations (CSPs) were calculated to identify residues that are involved in interacting with *Numb5.* Since the chemical shifts of seven residues I25, G27, W30, S61, R62, G65 and F97 were perturbed so significantly that the new locations cannot be determined, we acquired the ^15^N-edited NOESY-HSQC spectrum of MSI2-RRM1:*Numb5* complex, and assigned the backbone ^1^H and ^15^NH resonances of the seven residues in the bound state. CSPs were plotted against residue number (G21-K111) in Figure 3B. Residues exhibiting CSPs higher than one standard deviation above the average are G21, K22, F24, I25, G26, G27, S29, W30, Q31, T32, C50, S61, R62, G63, G65, F66, K94 V95 and F97. These nineteen residues interact directly or indirectly with the *Numb5* RNA oligomer.

### MSI2-RRM1 structure prediction

A model of MSI2-RRM1 was generated using CS-ROSETTA. This program utilizes protein backbone chemical shifts to select protein fragments from the protein data bank (PDB), followed by Rosetta Monte Carlo assembly and relaxation methods. The CS-ROSETTA program has been shown to be effective in *de novo* structure prediction for small proteins (≤16 kDa) [34, 35]. Residues in the N-terminus (M-3-K22) and C-terminus (A101-K111) are highly flexible and thus were excluded in the CS-ROSETTA calculation. A total of 25,000 structures were generated. The 10 lowest energy structures were selected and their averaged Cɑ root-mean-square-deviation (RMSD) against the lowest structure is 0.933 ± 0.284 Å. The superposition of the backbone atoms of the 10 lowest energy CS-ROSETTA structures and the ribbon diagram representation of the lowest energy structure for MSI2-RRM1 are shown in Figure 4A and 4B. The lowest energy structure is composed of five β-sheets (β1–β5) at residues 24–26, 47–52, 64–69, 84–86 and 89–96, and two ɑ helices (ɑ1 and ɑ2) at residues 34–43 and 73–81.

A homology model of MSI2-RRM1 was also constructed (Figure 4C). This model relies on the NMR structure of MSI1-RBD1: *Numb5* complex [26] as a template and the high sequence identity (80%) between MSI2-RRM1 and MSI1-RBD1. Similar to the CS-ROSETTA model, the homology model of MSI2-RRM1 also consists of five β-sheets (β1–β5) at residues 23–26, 47–52, 64–69, 84–86 and 89–95, and two ɑ helices (ɑ1 and ɑ2) at residues 33–42 and 72–82. The RMSD between the CS-ROSETTA model and homology model is 2.30 Å for 75 equivalent Cα atoms. Both the CS-ROSETTA model and the homology model exhibit a typical RNP-type fold consisting of four anti-parallel β-sheets (β1–β3 and β5) packed against two ɑ helices (ɑ1 and ɑ2), and another short β-sheet (β4) forming a β-turn with β5.

### RNA binding pocket

**Figure 6:**
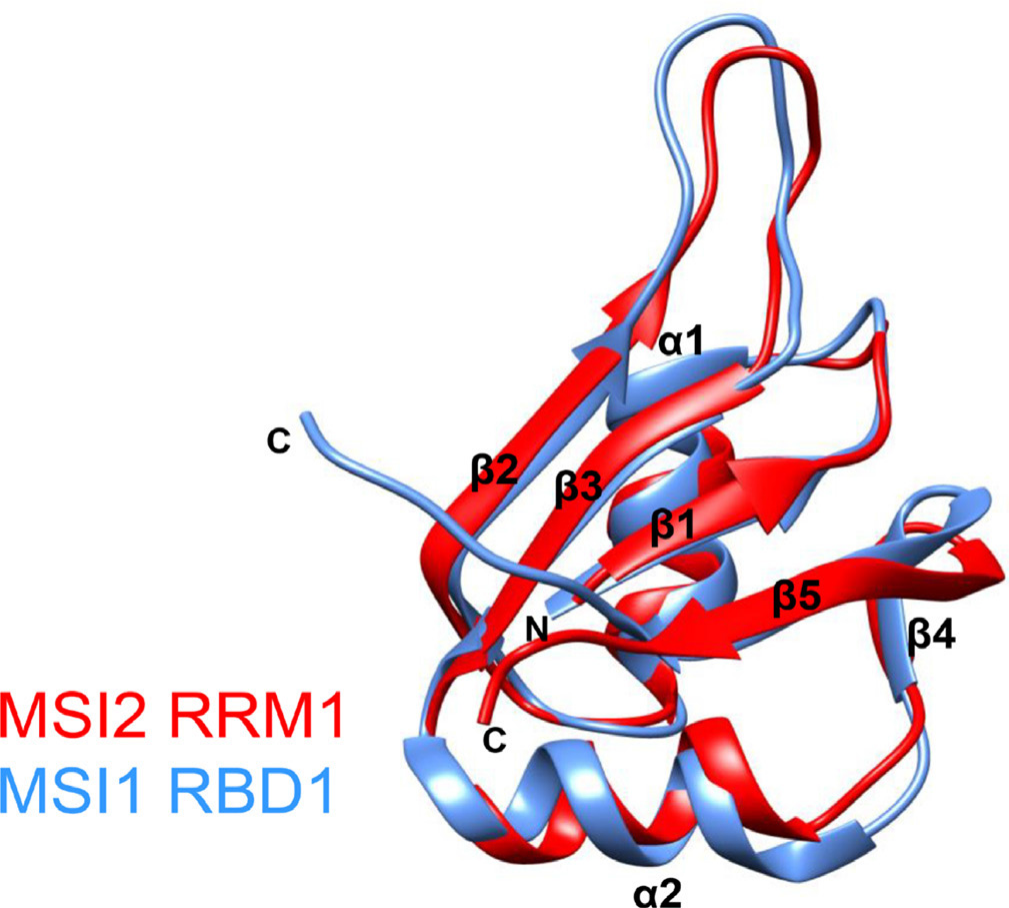
Overlay of the CS-ROSETTA model of MSI2-RRM1 (red) and the NMR structural model of MSI1-RBD1 (blue)

The agreement between the overall structures of the CS-ROSETTA model and the homology model of MSI2-RRM1 encouraged us to use the CS-ROSETTA model to identify the RNA binding pocket, and compare the binding pocket with that of MSI1-RBD1. Aligning the amino acid sequences of MSI2-RRM1 and MSI1-RBD1 and comparing their secondary structure elements revealed a high degree of secondary structure conservation between MSI2-RRM1 and MSI1-RBD1 (Figure 5A). Ohyama *et al.* performed an NMR titration of MSI1-RBD1 with *Numb5* [26]. Based on their results, we highlighted (in blue) seventeen residues (F23, I24, G25, G26, S28, W29, Q30, T31, M52, S60, R61, G62, G64, F65, K93, V94 and F96) in MSI1-RBD1 with largest CSPs on a ribbon diagram of the MSI1-RBD1 structure (Figure 5B right panel). Likewise, Figure 5B (left) shows in red seventeen residues (F24, I25, G26, G27, S29, W30, Q31, T32, C50, S61, R62, G63, G65, F66, K94, V95 and F97) that were significantly affected upon titration of *Numb5* in MSI2-RRM1. In the CS-ROSSETTA model of MSI2-RRM1, G21 and K22 are highly flexible, thus, residues G21 and K22 in MSI2-RRM1 and their equivalent residues C20 and K21 in MSI1-RBD1 were not mapped on to the ribbon diagram representations. In both proteins, the seventeen residues experiencing significant CSPs cluster around the central anti-parallel β-sheets and the surrounding loops. In MSI1-RBD1, this region is involved directly in binding with *Numb5* [26]. Therefore, we hypothesize that analogous to MSI1-RBD1, the RNA-binding pocket of MSI2-RRM1 is also composed of the central anti-parallel β-sheets and surrounding loops. In the surface representations, residues F24, G26, G27, S29, W30, Q31, R62, G63, F66, K94, V95 and F97 delineate the putative RNA-binding surface of MSI2 (Figure 5C left panel). Similarly, in MSI1-RBD1 F23, G25, G26, S28, W29, Q30, R61, G62, F65, K93, V94 and F96 lie along the RNA-binding surface where *Numb5* binds (Figure 5C right panel). Moreover, the CS-ROSETTA model of MSI2-RRM1 and the NMR structural model of MSI1-RBD1 are easily superimposed with an RMSD of 2.29 Å for 75 Cα (Figure 6). Taken together, our CSP mapping results suggest first, that specific interactions exist between MSI2-RRM1 and *Numb*5, and second, that the perturbed residues in MSI2-RRM1 are generally consistent with those in MSI1-RBD1, and finally, that MSI2-RRM1 and MS1-RBD1 have similar RNA-binding pocket.

## DISCUSSION

In this study, we characterized the MSI2-RRM1 protein and *Numb* RNA interactions using FP, TR-FRET and NMR, and revealed the key features of protein- RNA binding and the binding pocket. MSI2-RRM1 can specifically bind to *Numb5* to form a 1:1 complex, and the binding process exhibits a slow exchange behavior indicative of a high binding affinity. We propose that the putative binding pocket of MSI2-RRM1 is composed of the central anti-parallel β-sheets and the surrounding loops in the MSI2-RRM1 CS-ROSETTA model, which was supported by the fact that seventeen residues with significant CSPs (F24, I25, G26, G27, S29, W30, Q31, T32, C50, S61, R62, G63, G65, F66, K94, V95 and F97) cluster together and thus probably comprise the binding pocket. The putative RNA binding pocket of MSI2-MMR1 identified in the study contains the canonical RNP motifs RNP1 in β3 and RNP2 in β1 [36]. F64 and F66 in RNP1 and F24 in RNP2 are likely to canonically stack with RNA. The putative RNA binding pocket of MSI2-RRM1 also has unique features that W30 in the loop between β1 and α1 and F97 in the C-terminal end showed significant CSPs, and may involve in non-canonical base stacking interactions with RNA.

MSI2-RRM1 and MSI1-RBD1 have high similarity in their RNA-binding characteristics and RNA-binding pockets. Both proteins can specifically bind to *Numb5* in a 1:1 stoichiometric manner, and their bound and unbound states undergo slow exchange in the NMR chemical shift time scale. In both proteins, the residues involved in direct interaction with *Numb5* are generally consistent, moreover, seven residues in MSI2-RRM1 (I25, G27, W30, S61, R62, G65 and F97) whose chemical shifts were perturbed so significantly (ΔδH > 0.3 ppm) are all conserved in MSI1-RBD1, being I24, G26, W29, S60, R61, G64 and F96, and indeed they showed pronounced CSPs (ΔδH > 0.3 ppm) in MSI1-RBD1. These findings indicate both proteins use a similar set of residues to bind to *Numb5*. Their RNA-binding pockets are both formed by the central anti-parallel β-sheets and the surrounding loops.

The aromatic residues of MSI1-RBD1 that are directly involved in base stacking interactions with *Numb5* are F23, W29, F63, F65, and F96, these five residues are conserved in MSI2-RRM1, being F24, W30, F64, F66 and F97. Specifically, the aromatic rings of F23 and F96 interact with Ade3, W29 displayed a unique feature that its indole ring can stack with Gua1, F65 aromatic ring stacks with Gua4, and F63 interacts with Ura2 and Ade3. At molar ratio of MSI1-RBD1 to *Numb5* of 1, W29 and F96 exhibited pronounced CSPs (ΔδH > 0.5 ppm, ΔδN > 2.5 ppm), while F23, F63 and F65 experienced lower CSPs (ΔδH = ∼0.1 ppm, ΔδN = ∼0.5 ppm). Our CSPs analysis results revealed that titrating *Numb5* into MSI2-RRM1 induced significant CSPs for residues W30 and F97 (ΔδH > 0.5 ppm, ΔδN > 2.5 ppm), and F24, F64 and F66 exhibited lower CSPs in the range of 0.08–0.17 ppm. Therefore, the CSPs events observed here for F24, W30, F64, F66 and F97 in MSI2-RRM1, together with the high similarity between the overall foldings of MSI2-RRM1 and MSI1-RBD1, provide evidence that similar tryptophan-guanine, phenylalanine-adenine, phenylalanine-uracil and phenylalanine-guanine base stacking interactions are present in MSI2-RRM1.

Taken together, the existence of similarities in the CSPs of MSI2-RRM1 and MSI1-RBD1 due to RNA binding suggests the possibility of identifying novel small molecule inhibitors that are MSI2-specific as well as others that can function as MSI1/MSI2 dual inhibitors. Such inhibitors could potentially disrupt these unique MSI2-RNA aromatic amino acids mediated base stacking interactions or MSI-RNA interactions, thus leading to inhibition of MSI1/MSI2-mediated biological functions and compromising, among others, the viability of cancer cells that depend on MSI1/MSI2. Combined with our other efforts in discovering chemical probes, e.g. fragment based drug screening, obtaining the solution structure of MSI2-RRM1 will help the development of MSI1/2 inhibitors with structure-based rational design (Docking with NMR data) to discover compounds that fit the long narrow RNA-binding pocket of MSI2.

## MATERIALS AND METHODS

### Binding assays

*Numb* mRNA contains the Musashi recognition motif r(UAG), and is a binding target shared by both MSI1 and MSI2. In order to dissect the similarities and differences between MSI1 and MSI2 in the RNA-binding pocket, we used the same 15 nt *Numb* RNA (5′UAGGUAGUAGUUUUA) for our binding assays and the same 5nt *Numb* RNA (GUAGU) for the NMR studies as our previous study [33]. FP assay *was* carried out using a FITC (fluorescein isothiocyanate) labeled Numb RNA (*Numb*^*FITC*^: 5′UAGGUAGUAGUUUUA-FITC) according to our previous publication [33]. TR-FRET assay was carried out using Streptavidin-d2 beads (610SADLA, Cisbio, Bedford, MA) and MAb Anti-6HIS Tb cryptate Gold beads (61HI2TLA, Cisbio) in HTRF 96 well low volume plate (66PL96005, Cisbio) following the protocol recommended by the manufacturer. Fluorescent measurements were taken at room temperature using a BioTek Synergy H4 plate reader (Biotek, Winooski, VT).

### Protein expression and purification

The MSI2-RRM1 domain was sub-cloned into the pTGSG vector using the Ligation Independent Cloning method as described [37]. The non-labelled MSI2-RRM1 protein was expressed in *E. coli* BL21(DE3) pRARE and purified as previously described [33], and was kept in the buffer of 50 mM Tris-HCl, pH 8.0, 500 mM NaCl till use. The isotopically labelled protein was expressed in M9 medium using C^13^ glucose and N^15^ Ammonium Chloride as sole carbon and nitrogen source, respectively. The isotope uniformly labeled protein was isolated and purified using the same method as that of the unlabeled protein and then further purified through a Superdex™ 75 10/300 GL column equilibrated with 20 mM MES, pH 6.0, 150 mM NaCl. The protein was then concentrated and 5% D_2_O was added for NMR spectroscopy; Protein purity was assessed on coommassie stained acrylamide gel and protein concentrations were determined using the Bradford assay (Bio-Rad, Hercules, CA).

### NMR spectroscopy

All NMR experiments were performed at 298 K on a Bruker AVANCE 800 MHz spectrometer equipped with a triple resonance cryoprobe and a Bruker AVANCE 600 MHz spectrometer equipped with a triple resonance room temperature probe. The NMR buffer was 20 mM MES (pH 6.0), 150 mM NaCl and 5% D_2_O for spectrometer lock. NMR data were processed using the NMRPipe program [38] and visualized and analyzed using CCPN Analysis [39] and NMRViewJ [40].

Triple resonance NMR data were collected using ^13^C and ^15^N labeled MSI2-RRM1 samples in the range of 0.4–0.8 mM at volumes of 0.5–0.6 mL in standard 5 mm NMR tubes. Backbone resonance assignments were made by analyzing HNCA, HNCACB, HN(CO)CA, CBCACONH, HNCO and HN(CA)CO spectra [41–44]. ^15^N-etited NOESY-HSQC (120 ms mixing time) was collected using a sample containing 0.4 mM ^15^N labeled MSI2-RRM1 and 0.4 mM unlabeled *Numb5.*

^1^H-^15^N HSQC titration experiment was performed using 80 µM ^15^N-labeled MSI2-RRM1. Unlabeled r(GUAGU) (*Numb5*) stock solution was titrated into the MSI2-RRM1 sample to protein to ligand molar ratio of 1:0, 1:0.3,1:0.5, 1:0.7, 1:1 and 1:1.3. A ^1^H-^15^N HSQC spectrum was recorded for each titration point. Combined CSPs (Δδ) were calculated using the equation Δδ = [((ΔδH)^2^ + (ΔδN/5)^2^)/2]^1/2^. Where ΔδH and ΔδN are chemical shift changes of ^1^H and ^15^NH, respectively.

### CS-ROSETTA modeling and homology modeling

The CS-ROSETTA structures of MSI2-RRM1 (residues M23-R100) were calculated using the CS-ROSETTA server (https://csrosetta.bmrb.wisc.edu/csrosetta), utilizing the protein backbone ^1^H, ^15^NH, ^13^Cα and ^13^C’ chemical shifts of MSI2-RRM1 deposited in BMRB under accession code 27111. Twenty-four homologous sequences, including MSI1-RBD1 sequences (PDB IDs 1UAW and 2RS2), have PSI-BLAST e-scores < 0.05 and were excluded before the fragment search procedure. The homology model of MSI2-RRM1 was generated using the SWISS-MODEL server [45]. Residues G21 to K111 was aligned with the MSI1-RBD1 template (PDB ID 2RS2). The query and template sequence identity was 78%. The resulting model had a GMQE score of 0.65 and QMEAN score of –0.69.

## Abbreviations

(MSI): Musashi
(RRMs): RNA-recognition Motifs
(RBDs): RNA-binding Domains
(NMR): Nuclear Magnetic Resonance
(RBPs): RNA-binding proteins
(hnRNPs): heterogeneous nuclear ribonucleoproteins
(RNP): ribonucleoprotien
(FP): florescence polarization
(TR-FRET): time resolved Fluorescence Resonance Energy Transfer
(CSPs): Chemical shift perturbations
(PDB): protein data bank
(RMSD): root-mean-square-deviation
